# Re-establishing safer medical-circumcision-integrated initiation ceremonies for HIV prevention in a rural setting in Papua New Guinea. A multi-method acceptability study

**DOI:** 10.1371/journal.pone.0187577

**Published:** 2017-11-08

**Authors:** Clement Morris Manineng, David MacLaren, Maggie Baigry, Emil Trowalle, Reinhold Muller, Andrew Vallely, Patrick Gesch, Francis Hombhanje, William John McBride

**Affiliations:** 1 College of Medicine and Dentistry, James Cook University, Queensland, Australia; 2 Faculty of Medicine and Health Sciences, Divine Word University, Madang, Papua New Guinea; 3 East Sepik Provincial AIDS Committee, Wewak, Papua New Guinea; 4 Sexual and Reproductive Health Unit, Papua New Guinea Institute of Medical Research, Goroka, Papua New Guinea; Katholieke Universiteit Leuven Rega Institute for Medical Research, BELGIUM

## Abstract

**Background:**

Efforts to stem the spread of Human Immunodeficiency Virus (HIV) in Papua New Guinea (PNG) are hampered by multiple interrelated factors including limited health services, extreme diversities in culture and language and highly prevalent gender inequity, domestic violence and poverty. In the rural district of Yangoru-Saussia, a revival of previously ceased male initiation ceremonies (MICs) is being considered for a comprehensive approach to HIV prevention. In this study, we explore the local acceptability of this undertaking including replacing traditional penile cutting practices with medical male circumcision (MMC).

**Methods:**

A multi-method study comprising three phases. Phase one, focus group discussions with male elders to explore locally appropriate approaches to HIV prevention; Phase two, interviews and a cross-sectional survey with community men and women to assess views on MICs that include MMC for HIV prevention; Phase three, interviews with cultural leaders and a cross sectional survey to assess the acceptability of replacing traditional penile bleeding with MMC.

**Results:**

Cultural leaders expressed that re-establishing MICs was locally appropriate for HIV prevention given the focus on character building and cultural preservation. Most surveyed participants (81.5%) supported re-establishing MICs and 92.2% supported adapting MICs with MMC. Changes to penile bleeding emerged as a contentious and contested issue given its cultural significance in symbolizing initiates’ transition from childhood to adulthood. Participants were concerned about potential clash with modern education, introduced religious beliefs and limited government support in leadership and funding.

**Conclusions:**

Most people in this study in Yangoru-Saussia support re-establishing MICs and replacing traditional penile bleeding with MMC. This culturally-sensitive alignment of MMC (and HIV prevention) with revived MICs responds to a national health priority in PNG and acts as an example of providing culturally-sensitive male circumcision for HIV prevention recommended by WHO/UNAIDS. However, the implementation of this undertaking will require considerable effort, especially when modern pursuits in education and religion must be factored and when there is expectation for local authorities to lead and provide funding.

## Background

Papua New Guinea (PNG) has the highest Human Immunodeficiency Virus (HIV) prevalence in the Western Pacific with nearly 1% of the adult population infected [1]. Available data suggests that the epidemic is concentrated rather than generalized, reflecting higher prevalence in urban centres, highlands provinces and among high risk-groups including sex-workers [2–4]. However, national and international health experts warn that HIV is still a critical threat in the general PNG population [3, 5]. Innovative but context-specific approaches are urgently required to limit HIV spread in this setting.

HIV prevention in PNG has had impediments on many fronts. PNG has over 800 ethnic groups, some 850 indigenous languages, multiple religious denominations and rapidly changing socio-economic and political environments [6–8]. On top of that, 85% of the population lead tribal lives in isolated rural communities where essential services including basic health care and health promotion services are barely functional or non-existent. Where such services exist, their uptake and use is often hampered by low literacy levels and language barriers [6, 7, 9]. Furthermore, the negative impacts of rapid socioeconomic developments including destabilization of traditional social structures and value systems are contributing to high levels of gender inequity, domestic violence, poverty and corruption, all of which have interwoven effects on escalating HIV transmission in PNG [10–15].

Initiation ceremonies or transition rites have been important traditional activities that have contributed to community well-being in some parts of PNG (as elsewhere) for millennia [16–22]. These ceremonies were vital particularly in precolonial days to guide young adults through their turbulent adolescent stages and enabling them to discard carefree childhood behaviours and adopt adulthood responsibilities [19]. Adult responsibilities include active participation in gender specific roles, living independent lives and respecting established social structures and customary norms. However, these traditional behaviour-guiding practices have struggled to continue alongside modern interests in career-driven education, introduced Christianity and financial freedom [22–29]. Consequent discontinuation of initiation ceremonies meant that an important avenue for behavioural guidance was denied to adolescent boys and girls in this setting.

An undertaking to re-establish male initiation ceremonies (MICs) is underway in Yangoru-Saussia, a rural district in East Sepik Province, PNG [30]. Re-establishing MICs may be difficult considering that such a venture would require reviving previously abandoned practices, some of which are conflicting with Christianity–the dominant religion. In addition, local people, in their contemporary livelihood may not be willing to accommodate the requirements of another program in their daily routines, particularly if that program is deemed to be contradicting current efforts in pushing for modernization. Moreover, previously abandoned MICs in Yangoru-Saussia included high-risk penile-bleeding operations [18] which would need to be replaced with medical male circumcision (MMC), a safer modern alternative. However, these penile operations have significant cultural meaning including symbolising transition from childhood to adulthood [31–34]. Thus, there are significant questions that need to be answered before progress can be made towards establishing a MIC that could comprise substituting traditional penile-bleeding with modern MMC.

Replacing traditional penile-bleeding procedures with MMC could provide attractive health benefits to initiates. In the short term, substitution with MMC may prevent adverse events and reduce health risks to individual participants who would otherwise be subjected to the high-risk traditional penile-bleeding procedure. In the long term, the health benefits could include prevention of Human Immunodeficiency Virus (HIV)–as demonstrated by three randomized controlled trials [35–37]–and lowering disease risks (including of certain cancers) related to Human Papilloma Virus (HPV) [38–40]. Furthermore, if MMC at MICs could be complemented with HIV counselling and testing, a comprehensive approach to HIV prevention and a culturally relevant public health program could be realized in this contemporary setting.

Re-establishing MICs could provide attractive benefits to local communities and to health service providers. To local communities, re-establishing MICs means that adolescent boys in the communities may now have access to previously discontinued traditional process of behavioural mentoring. Local communities also stand to benefit through the revitalization of cultural practices (linked to MICs) including unique artwork and traditional dances [30]. To health service providers, re-established MICs may offer two main benefits; one: community ownership and enhanced participation (because of local relevance) could lead to better outcomes in preventing adverse events and controlling HIV [41–43]; two: cost to health program budget could be minimal given that custom requires initiates and their supporters to pay for their participation. In this part of the pre-colonial world and like initiating communities across Melanesia, participation at initiation ceremonies were associated with power and dominance–traits that cannot be gained without first sacrificing or depositing something of monetary value [44–46].

The purpose of this study therefore, was to assess the acceptability of re-establishing MICs, including replacing traditional penile bleeding procedures with MMC for HIV prevention in Yangoru-Saussia, PNG. In this paper, we present the results of this acceptability assessment and discuss the implications in relation to a comprehensive approach to HIV prevention in this setting in PNG.

## Method

### Study design and setting

To assess the acceptability and feasibility of integrating medical male circumcision (MMC) into male initiation ceremonies (MICs) in Yangoru-Saussia district of East Sepik Province, PNG, a multi-method study was conducted in three phases.

Phase one: Focus group discussions (FGDs). In 2009, four FGDs were conducted with male elders representing each of the four government divisions or Local Level Government (LLG) areas: Numbo, Sausse, East Yangoru and West Yangoru. FGDs were conducted at respective LLG council chambers. The main aim of phase one was to gauge cultural leaders’ views on most appropriate local approaches for HIV prevention in Yangoru-Saussia district of East Sepik Province. Male leaders were first to air their views in phase one in accordance with the established patriarchal social structure [46].

Phase two: Key-informant interviews and one cross-sectional survey. In 2011, sixteen cultural leaders were interviewed and 200 people completed survey questions. Interviews and surveys were conducted at public gatherings including roadside markets (along the Sepik Highway), at villages and at or near schools and clinics. The main aim of phase two was to investigate the local peoples’ views on reviving MICs for HIV prevention. Both males and females participated in this phase.

Phase three: Key-informant interviews and one cross-sectional survey. In 2015, ten cultural leaders were interviewed and 64 people completed survey questions. Similar to phase two, interviews and surveys were conducted at public gatherings including roadside markets (along the Sepik Highway), at villages and at or near schools and clinics. The main aim of phase three was to assess the acceptability of replacing traditional penile bleeding with MMC at future MICs. Both males and females participated in the survey but only men participated in the individual interviews because female cultural leaders stated that MICs was a male affair and should be discussed with men.

### Participant recruitment

Purposive sampling was applied to recruit participants into FGDs (phase one) and key-informant interviews (phases two and three) [47]. A total of 40 men (10 per LLG) participated in the FGDs while 16 and 10 participants were interviewed (by same gender researchers) during phases two and three key-informant interviews. Pre-identified participants with specific cultural knowledge and community leadership positions were approached in a respectful manner and requested to share their views at FGDs or individual interviews. For FGDs, participants gathered at their respective LLG council chambers on specified dates and times. Individual interviews were conducted at a time and place convenient to participants. Prior to interviews and discussions, all participants had the study explained to them and completed informed consent procedures.

Convenience sampling was applied to recruit participants for the cross-sectional surveys [47]. Phase two, 200 participants completed surveys while phase three, 64 completed surveys. Trained male and female research assistants helped administer the questionnaires (some were self-administered) to respective gender participants at roadside markets (along the Sepik Highway) and at or near health and education facilities. Persons aged 16 years (accepted as young adults in this setting) and above were approached casually and requested to share their views. People who appeared ill or mentally in-capacitated were not included. Consent procedures were conducted prior to participation.

### Data collection

The FGDs (phase one) were facilitated using a specifically designed discussion guide (see S1 Appendix). This discussion guide had open-ended questions about ‘past and present traditional practices’; ‘HIV transmission’; and ‘best approaches to HIV prevention in local communities’. Trained male research assistants facilitated the discussions in Tok-Pisin, the local lingua-franca. The voice-recorded discussions were transcribed verbatim, translated into English and stored on password protected data storing devices.

The key-informant interviews (phases two and three) were conducted using interview guides designed from information generated by FGDs (in phase one). The phase two interview guide (see S2 Appendix) had open-ended questions that prompted for detailed descriptions of initiation ceremonies and clarifications on how initiation ceremonies could help prevent HIV in local communities. The phase three interview guide (see S5 Appendix) had open-ended questions about the possibility of including MMC at future MICs. The voice-recorded interviews (facilitated in Tok-Pisin) were transcribed verbatim, translated and stored on the devices containing FGD data.

The cross-sectional surveys (phases two and three) were conducted using structured survey forms that contained both open-ended and closed-ended questions (see S3 Appendix and S4 Appendix). For each survey, the generated information was double entered onto Microsoft Excel files, cleaned (using the ‘sort and filter’ function) and stored with the rest of the research data.

### Data analysis

Qualitative data generated from FGDs, key-informant interviews and responses to ‘open-ended questions’ in surveys were analysed manually using thematic analysis. Transcribed data was colour coded and grouped initially into categories. The categorized data were then inserted under respective linking themes on a theme table and inductive and adductive thought processes were applied to weave the data into a cohesive storyline [48, 49].

Quantitative data analysis was done on SPSS version 22. Each data set (see S1 File and S2 File) from cross-sectional surveys (phases one and two) was analysed separately. Prior to analysis, data in each cleaned data set had value labels applied. Bivariate association tests between categorical variables were done using unpaired Classical Chi-square test [50, 51].

### Ethical considerations

All study participants provided informed written consents prior to participating in the study. Participants 16–17 years of age (recruited in the cross-sectional surveys) were regarded in the local cultural context as young adults and were allowed to provide their own informed written consents. These ethical procedures were reviewed and endorsed by Divine Word University Research Ethics Committee–approval date: 10^th^ November 2009 (phase one); PNG National AIDS Council Research Advisory Committee grant RES10 026 (phase two) and PNG Medical Research Advisory Committee grant MRAC 14.33 and James Cook University Research Ethics Committee grant H6006 (phase three).

## Results

Most cultural leaders interviewed in phase one of this study expressed a desire to have a socio-cultural approach to HIV prevention that reflected local history and culture in Yangoru-Saussia. Initiation ceremonies (including female ceremonies) were depicted as mediums for education (pre-colonial schools) that had an emphasis on character building. Leaders stated that there was a general deficiency in moral standards in contemporary communities. Initiation ceremonies were seen to be able to instil positive values to initiates to improve their lives, their health and thereby protect them and their families from HIV. Not only this, but those traditions were an excellent way to preserve local cultural practices and male initiation ceremonies (MICs) were therefore further investigated in this study.

### Subjects and activities of the old male initiation ceremonies (from phases one and two)

Local cultural leaders reported that the old MICs could last from 3 months to a year or more and was guided by two major objectives. The first objective was to transfer existing traditional knowledge and skills from old generation men to new generation men, and the second was to toughen initiates’ physical, emotional and mental faculties. The latter was basically an opportunity for initiates to prove their worth as capable men: men able to withstand pain and undesirable emotions.

To achieve the first objective, leaders described that cultural subjects were taught at secluded men-only quarters commonly referred to as ‘hausman’ or ‘hausboy’. The subjects taught included *‘Miye-Hru Miyekwo’* (Garamut or slit-gong drum communication), *‘Lomo-Hangu’* (traditional song and dance), *‘Pilanang-Rhambanang’* (Arts and Craft), *‘Paiye-Nangri’* (Public Speaking), *Maiye-Tachk* (Witchcraft and Sorcery). During the process of mastering the above subjects, the initiates also learnt about their spirit totems, customary relationships and obligations and land boundaries and potential conflicts. To achieve the second objective, leaders explained that the initiates had to endure several trials. Initial trial was separation from family especially parents and siblings. At some stages, the initiates go without food and water and are brought into the *huelombo ka* (spirit house). Towards the end of the ceremony the initiates were reported to be thrashed with fresh sticks, rubbed with stinging nettles and the penis cut to cause bleeding.

According to local cultural leaders, penile bleeding happened at a designated spot in a stream. A sharp object such as a cassowary bone was used to split open the glans from the urethral end. The consequent pain and bleeding was described as being symbolic of severing an initiate’s ties with his mother and therefore his childhood. Cultural leaders interviewed were not able to specify the amount of blood lost because the released blood (which is considered as old or waste blood) went into the stream and was washed away. However, cultural leaders recalled that blood loss was substantial. The effect of releasing this old or waste blood was said (by some cultural leaders) to make a man feel energetic and appear lighter in complexion.

“Yeah a great deal (of blood-loss). It’s this old, waste blood that will be released. Two to three days after releasing this waste blood, you will see a lightness in your skin and you will be smart in anything you want to do, play soccer or fight, anything; it’s just normal to you” Male leader–West Yangoru LLG.

### Motivators to revive male initiation ceremonies (from phases one, two and three)

The desire to revive MICs was high in those interviewed. Participants generally spoke with respect and renown of the past initiation ceremonies and shared insights of the courage, strength and wisdom that come with it. Participants stated that initiated men (and women) had a heightened ability to reason and discern between ‘right’ and ‘wrong’ and that they demonstrated the strength and courage to choose and do that which was ‘right’. This ability for men to choose to do ‘right’ according to cultural leaders was how HIV could be prevented through MICs. Initiated men were said to take more responsibility for their actions including limiting sex to marital relationships and therefore reducing the risk of acquiring HIV. The participants highlighted that this quality (of wisdom and integrity) was missing in current times. Many participants, even those with high level education and regular church goers, viewed ‘a future revival of MICs’ as an opportunity to re-establish the old source of wisdom and integrity to complement existing value systems. Study participants also pointed out that many challenges currently faced in the communities, including law and order, domestic violence and HIV/AIDS could be addressed through MICs. Revival of MICs was therefore seen to have the potential to facilitate safe, healthy and prosperous communities in Yangoru-Saussia, in accordance with the national development strategy–PNG Vision 2050 [52]. A prominent cultural leader summarised the desire to revive MICs with this metaphorical remark:

“A revival of male initiation ceremonies is not wrong, it is the right thing to do and many people are waiting for someone to start it. Put the fire at one spot and everywhere in Yangoru-Saussia will come alight with the fires of initiation ceremonies”. Male cultural leader–East Yangoru LLG.

### Subjects and activities to be excluded or modified (from phase three)

Several cultural activities of the past MICs were deemed to be unsuitable or of little use for modern times by the study participants, and therefore needed to be excluded or modified. Just about all participants had serious concerns with the possibility of including sorcery and *Sanguma* (malicious witchcraft) as subjects at future MICs. It was emphasised that a revival of the old MICs should focus on elements of past programs that benefited the wider community rather than on elements that brought harm on individuals and their families. Most illnesses and deaths of the past and even of current times were attributed to sorcery and witchcraft and any revival of those old malicious elements, most people viewed, could receive unrelenting opposition from many parties including, churches, local authorities and potential participants. While people spoke earnestly about the need to re-establish the native tradition, there was obvious hesitation at the perceived possibility of reviving sorcery and *Sanguma* concomitantly with the MICs.

Most study participants were open to suggestions to increase participant safety by substituting penile bleeding with MMC. However, not all participants supported the suggestion to completely exclude penile bleeding from MICs. There were some cultural leaders who showed hesitation and indirect disapproval. Penile bleeding was highlighted by many respondents as being important or central to MICs. In other words, complete exclusion of penile bleeding from MICs could potentially decrease the meaning and cultural significance of the entire initiation process. In realizing this significance, some cultural leaders pointed out that some men today use razor blades to make small nicks on the glans penis to release small amounts of blood. Some participants suggested future MICs adopt this milder form of penile bleeding.

“Nowadays, some men bleed the penis with razor blades. We could do the same for initiates in the new program. Take them to the river and using a razor blade, make a small cut on the opening of the penile urethra and press so that blood shoots out”. Male cultural leader–East Yangoru LLG.

One interviewed female participant was direct with her disagreement on the possibility of modifying traditional MICs and of involving non-indigenous health workers to perform MMC at traditional male initiation ceremonies.

“Culture must be original. Do not bring in outsiders such as health workers into the traditional culture”. Female respondent–East Yangoru LLG (response from CSS).

### Potential barriers: Few culture experts, lack in government support and limited native language use (from phases two and three)

Many respondents stated that local expertise on specific elements of the MICs was lacking and provided suggestions to cater for this deficiency. Some respondents pointed out that there were at least one or two people in the communities who had enough expertise to lead a male initiation process. Interviewed cultural leaders suggested for expert or senior cultural leaders to move between initiating communities to ensure things are done in accordance with local tradition. Regarding the teaching of individual cultural subjects, study participants indicated that there were many local cultural leaders who had shown interest to participate as teachers. Some of these men had teaching and music backgrounds and were said to have the skills to facilitate easier and better learning experience for initiates. It was explained, for example that someone with music background might be able to structure the *garamut* beats into teachable codes and help initiates to grasp the beats and rhythms faster.

Some cultural leaders reported that they struggled to continue work on local culture because government was not supportive. These cultural leaders have on many occasions attempted to get the government to support their culture programs but almost all these attempts have been unsuccessful.

“I already started culture work but we could not maintain or progress that work because we see that there is no support from the government. Ok I have a number of custom (leaders) who are now with me (he names some of the leaders), they are ready to work but there is no one to lead and provide support so work on culture could continue”. Male cultural leader–Numbo LLG.

One of the pressing issues highlighted by local leaders was the use of local vernacular. The expert use of local native language was said to be necessary for successful facilitation of learning in matters to do with local culture. There was a general concern that while most young people can hear and understand, they cannot speak or fully express themselves in the native language. A reputable leader reasoned that the transmission of indigenous cultural knowledge between old and young generation depended very much on the language of instruction and that most matters to do with indigenous culture were intrinsically connected with the native language.

“But very important…they will not beat the garamut, our sons, they will not be able to do Paiye-Nangri (public speaking in local vernacular), they will not be able to ah…perform the Lomo-hangu. They must first know our tokples (native language). When they know the tokples, they will be able to beat the garamut …If not, just like me, they will find it difficult”. Community leader–West Yangoru.

Cultural leaders further expressed that, native language was dying because opportunities to engage in it were very limited and often confined to rare ceremonial activities. It was emphasized that individuals need to use local vernacular in their everyday vocabulary in order to ensure its survival. From this reasoning, interviewed leaders suggested that all verbal communications within initiation grounds be restricted to local vernacular. This restriction, participants thought, would help familiarise initiates with their native language and will also ease the facilitation of knowledge and skills transmission at future MICs.

### Most survey participants supported revival of male initiation ceremonies and inclusion of medical male circumcision (from phases two and three)

One of the key questions in the phase-two cross-sectional survey was “would you support revival of male initiation ceremonies?” This survey had 200 participants– 101 (50.5%) male and 99 (49.5%) female. Median age was 36 (inter-quartile range 25–47.75). Overall, 81.5% (n = 163) supported the proposition to revive MICs. Frequency of responses by demographic characteristics shown in Table 1.

**Table 1:** Reponses by demographic characteristics (phase two cross-sectional survey).

Would you support revival of male initiation ceremonies?				
	Yes % (n)	No % (n)	Total (n)	p-value
Total	81.5% (163)	18.5% (37)	200	
Data collect mode				
Interview	85.1% (114)	14.9% (20)	134	0.064
Self-administered	74.2% (49)	25.8% (17)	66	
Gender				
Male	90.1% (91)	9.9% (10)	101	0.002
Female	72.7% (72)	27.3% (27)	99	
Age group				
16–25	71.2% (37)	28.8% (15)	52	0.170
26–35	84.2% (32)	15.8% (6)	38	
36–45	85.1% (40)	14.9% (7)	47	
46+	86.3% (44)	13.7% (7)	51	
Marital status				
Married	86.1% (118)	13.9% (19)	137	0.130
Single	71.2% (42)	28.8% (17)	59	
LLG of origin				
Numbo	89.8% (44)	10.2% (5)	49	0.075
Sausse	70.0% (35)	30.0% (15)	50	
East Yangoru	82.0% (41)	18.0% (9)	50	
West Yangoru	84.3% (43)	15.7% (8)	51	
Education level				
Up to high School	81.3% (100)	17.9% (23)	123	0.927
Up to tertiary school	81.8% (63)	18.2% (14)	77	
Church affiliation				
Catholic	82.8% (77)	17.2% (16)	93	0.748
Assemblies of God (AOG)	84.0% (42)	16.0% (8)	50	
Seventh Day Adventist (SDA)	75.7% (28)	24.3% (9)	37	
Other	78.9% (15)	21.1% (4)	19	
Initiation status				
Initiated	83.5% (81)	16.5% (16)	97	0.503
Not initiated	79.8% (79)	20.2% (20)	99	

One of the key questions in the phase-three cross-sectional survey was “would you support including medical male circumcision at male initiation ceremonies?” This survey had 64 participants– 36 (56.3%) male and 28 (43.7%) female. Median age was 40.5 (inter-quartile range 30.25–49.74). Overall, 92.2% (n = 59) supported the proposition to include MMC at revived MICs. Frequency of responses by demographic characteristics shown in Table 2.

**Table 2:** Responses by demographic characteristics (Phase three cross-sectional survey).

Would you support including medical male circumcision at male initiation ceremonies?				
	Yes % (n)	No % (n)	Total (n)	p-value
Total	92.2% (59)	7.8% (5)	64	
Data collect mode				
Interview	90.2% (37)	9.8% (4)	41	0.439
Self-administered	95.7% (22)	4.3% (1)	23	
Gender				
Male	91.7% (33)	8.3% (3)	36	0.860
Female	92.9% (26)	7.1% (2)	28	
Age group				
17–25	90.0% (9)	10.0% (1)	10	0.325
26–35	93.3% (14)	6.7% (1)	15	
36–45	100.0% (20)	0.0% (0)	20	
46+	84.2% (16)	15.8% (3)	19	
Marital status				
Married	92.6% (50)	7.4% (4)	54	0.779
Single	90.0% (9)	10.0% (1)	10	
LLG of origin				
Numbo	91.3% (21)	8.7% (2)	23	0.840
Sausse	100.0% (7)	0.0% (0)	7	
East Yangoru	89.5% (17)	10.5% (2)	19	
West Yangoru	93.3% (14)	6.7% (1)	15	
Education level				
Up to high school	93.0% (53)	7.0% (4)	57	0.499
Up to tertiary school	85.7% (6)	14.3% (1)	7	
Church affiliation				
Catholic	92.3% (36)	7.7% (3)	39	0.686
Assemblies of God (AOG)	100.0% (8)	0.0% (0)	8	
Seventh Day Adventist (SDA)	86.7% (13)	13.3% (2)	15	
Revival	100.0% (2)	0.0% (0)	2	
Initiation status				
Initiated	91.2% (31)	8.8% (3)	34	0.748
Not initiated	93.3% (28)	6.7% (2)	30	

## Discussion

Re-establishing previously ceased traditional practices and adapting them with modern alternatives to address contemporary health and sociocultural needs is a relatively new area of investigation. Information presented can be of significant benefit to policy makers across many disciplines. This study to assess the acceptability of reviving and modifying male initiation ceremonies (MICs) for a comprehensive approach to HIV prevention is the first such study in Papua New Guinea (PNG). Results demonstrate a positive attitude towards establishing a safer version of the pre-colonial MICs in Yangoru-Saussia. Results also expand evidence for local, national and international policy makers to support locally appropriate HIV prevention strategies in PNG and other culturally, linguistically and geographically diverse nations [9, 14, 31, 33, 34, 41, 42, 53–57].

Overall, participants favoured the establishment of a modified version (that includes medical male circumcision) of the previously ceased MICs. Most participants (81.5%) supported the proposition to re-establish MICs and 92.2% approved inclusion of medical male circumcision (MMC). Participants consistently expressed an appreciation of traditional practices and the value of these practices for contemporary living. Although initiation ceremonies have now ceased, almost half of the survey participants were initiated, demonstrating existence of considerable experience and knowledge of past practices in the district. Cultural leaders depicted MICs as pre-colonial schools with an emphasis on character building. MICs were therefore seen to address a perceived deficiency in moral standards in contemporary communities and be a base for preventing HIV and other social challenges.

Support for the revival of MICs was consistently high across different sectors and characteristics of study participants. The only statistically significant difference was in gender with fewer females supporting the revival of MICs than men (72.7% vs 90.1% p = 0.002). However, the majority of women still supported the idea. Survey participants interviewed by researchers (because of low-literacy) were more likely to support the revival of MICs compared to those who self-administered the survey (85.1% vs 74.2% p = 0.064). However, both groups overwhelmingly supported the notion to revive MICs. Although not statistically significant, fewer young people, singles, Seventh Day Adventist (SDA) church affiliates and participants of Sausse local level government areas (LLG) supported revival of MICs. Lower support from young people may reflect less life experience and consequent limited understanding of MICs and the objective of reviving MICs. Conversely, lower support could portray an inclination towards modern lifestyles given the younger generations’ greater exposure (compared to older generation) to modern schools and modern influences. Younger generations’ lower support could also reflect their indifference towards a practice that may place them at risk of physical and emotional trauma inflicted by older men. Lower support from SDA affiliated participants may reflect the church’s introduced conservative Christian religious beliefs and general reticence to support local cultural traditions [58]. Cultural practices of some villages on the plains in Sausse LLG are closer to Sepik River practices than to the rest of Yangoru-Saussia and may explain lower support (70.0%) compared to other LLGs (82.0%, 84.3%, 89.8%). In addition, some villages used sharp objects like cassowary bones to split the glans penis as outlined by our study participants while other villages used barbed bush vines to bleed the penis in a ‘bottle-brushing’ fashion as described by Tuzin (1980)[22]. Future research could include mapping the type of penile bleeding practices in Yangoru-Saussia and their influence on an undertaking to revive MICs.

The call for government support by some cultural leaders may actually reflect perceived barriers in structuring and financing cultural programs including MICs. In terms of finance, there could be expectations of free or government sponsored MICs and it is paramount that any finance-related communication between program planners and cultural leaders (and members of their communities) be based on replicating the ‘user-pay’ arrangements of the precolonial MICs. It must be emphasized that custom requires participants to contribute to staging MICs. Custom also obligates participants to give back something of monetary value to those who facilitate the initiation process. Thus, without a ‘user-pay’ arrangement, re-established MICs could lose their customary worth and significance. Regarding program structure, it is important to note that the only time available for in-school boys to participate at MICs are the official school holidays, the sum in one academic year of which is just under three months [59]. This essentially means that the initiation process that previously took 3–12 months should be accelerated. Otherwise, program planners could consider omitting ceremonial activities that appear redundant or are conflicting with modern world views. Similarly, there is need for local authorities to articulate–perhaps in a clear policy direction–a conceptual and implementation framework for cultural activities including the potential re-establishment of MICs in the district.

Sorcery and *Sanguma* (malicious witchcraft) were of major concern to participants and were named as aspects of the old MICs that needed to be excluded. Sorcery and *Sanguma* herein referred to as ‘black magic’[60] are activities that participants said did not reflect contemporary values and therefore could not be included in revived MICs. In Yangoru-Saussia, most deaths and ailments are perceived to be related–even in current times–to black magic [61, 62]. Given this prevalent perception on the connection between black magic and adversities, some participants spoke against the revival of MICs because of their belief that black magic practices were to be included in the new program. Consequently, if the new program is perceived as a potential place for black magic to be strengthened, it was clear that most people would stop their young men from participating. Furthermore, most local people affiliate with Christian church denominations and oppose activities that support black magic or that challenge Christian principles. Any undertaking to revive MICs would have greater acceptance if it is communicated that black magic would not be included.

Local people in Yangoru-Saussia are custodians of their own traditional practices and as such, their views and opinions matter to future cultural programs. In addition, incorporating local views and suggestions into future MICs is vital because it is the local people who will design, adopt and implement any modifications to their contemporary society. Furthermore, including local people in this process reinforces respect and underpins their leadership, support and participation at future programs [41, 63, 64]. Similarly, it is essential that culturally sensitive modifications be carefully considered within revived MICs. Without this, the venture risks being meaningless to the very people it was intended to benefit. Therefore, a culturally sensitive inclusion of MMC at revived MICs, to replace foreskin cutting and penile bleeding procedures at traditional MICs, would require surgical procedures that usually occur in health facilities to happen at the ceremonial grounds. Further, health workers would need to be from the local area and they should partake in the ritual restrictions as required by custom [65]. This culturally sensitive alignment of MMC with a local cultural program is an excellent example of providing culturally specific male circumcision for HIV prevention as recommended by World Health Organization (WHO) and United Nations Program for HIV/AIDS (UNAIDS) [66]. Likewise, establishing a contemporary version of MICs will be in-line with a joint United Nations Educational, Scientific and Cultural Organization (UNESCO) and UNAIDS recommendation for HIV/AIDS education to include the teaching of life-skills and balanced gender roles [67]. Moreover, aligning MMC with a local cultural program would fulfil a PNG health priority for MMC to be made accessible for men who undergo high-risk foreskin cutting at non-clinical settings in PNG [68–70].

Penile bleeding is a culturally significant ritual at MICs in Yangoru-Saussia and emerged as a contentious and contested issue in this study. Some participants were happy that old penile bleeding procedures be replaced by MMC, while others were not. The loss of blood through the penile bleeding process is symbolic of initiates severing maternal ties (and therefore their childhood) and transitioning into adulthood. It is therefore important to understand both natures of blood loss: the procedural and symbolic. That is, while MMC may provide a ‘bio-medically safer’ option than using a cassowary bone during MICs, it may not satisfy the symbolic significance of disconnecting with childhood where pain (both physical and emotional) and blood loss is expected. In acknowledging the latter, some cultural leaders indicated that penile bleeding at future MICs could continue even if MMC were to be included. This fact points to the challenge of including modern alternatives without compromising the cultural meaning and significance of MICs in this setting. If this is the case and penile bleeding, even in its mild contemporary form (with use of razor blades) takes place at future MICs, the objective of minimizing health risks including HIV prevention could be undermined. Hence, the possibility of initiates undergoing razor-blade-induced penile bleeding must be considered and accommodated in risk-reduction plans (including for HIV prevention) for future programs.

There are limitations to this study that should be noted. Firstly, the findings and implications presented are specific to Yangoru-Saussia district and cannot be extrapolated to other settings in PNG. Yet, the rural contemporary setting in Yangoru-Saussia and the issues discussed are quite similar to other initiating indigenous communities striving to counter the side-effects of rapid modernization on local cultural practices and values. Secondly, it is acknowledged that local researchers including CM belong to the district and culture under study and raises the possibility of participant and researcher bias. Conversely, researchers’ indigenous, education and health professional backgrounds may have fostered trust between participants and research team, resulting in open and honest responses [71]. Thirdly, the application of convenience sampling means that views captured in this study may not be representative of all people in Yangoru-Saussia. However, a reasonable representation of local peoples’ views may have been captured given that people in Yangoru-Saussia belong to a society that prioritises collective pursuits over individual pursuits and regularly congregate at popular meeting venues such as markets, health centres, schools and conference chambers.

Future studies should further investigate results presented in this paper. Differences in support for revived MICs by men and women should be explored. Many cultural leaders expect government support for the revival and maintenance of their native cultural practices. Subsequent enquiry should investigate cultural leader expectations and provide an assessment on the practicality or otherwise of reviving MMC-integrated MICs with or without government support. In addition, the health benefits of penile blood-letting claimed by some participants of this study could be an interesting area for further studies. For instance, the health benefits claimed could be related to therapeutic phlebotomy, although the point of blood-letting is different. Therapeutic phlebotomy refers to deliberate release of blood (usually from external veins) to treat certain medical conditions. This therapeutic option is being investigated for its potential in reducing blood viscosity and enhancing oxygen circulation and tissue perfusion [72–74]. It is also important to note that the local native language has become second-place to introduced languages, local culture experts are too few and government support is lacking. Moreover, program planners should note that older men in this setting could use MICs to place adolescent boys under duress and every effort must be made to ensure that future MICs are free of coercion, violence and abuse. These multiple and interlinked issues all need to be carefully considered in the undertaking to re-establish safer MICs in Yangoru-Saussia, PNG. Insights to these issues will emerge through ongoing research by CM and his team of local and international researchers over the coming years.

## Conclusions

This study broadens evidence supporting locally appropriate HIV interventions in PNG and other diverse cultural, linguistic and geographical settings. Most people in this study in Yangoru-Saussia accept safer MMC-integrated MICs as a viable option for their contemporary society given the focus on character building and cultural preservation. However, implementation of modified MICs will require considerable effort, especially when modern education, introduced religion, contemporary economic pursuits and abuse prevention must be incorporated. Similarly, penile bleeding is a culturally significant ritual and emerged as a contentious and contested issue in this study. Allowing blood loss at initiation is thought by some to help a boy transition into a man and needs to be carefully considered in future programs. A culturally sensitive alignment of MMC with a proposed local cultural program is an example of providing culturally-specific male circumcision for HIV prevention as recommended by WHO/UNAIDS. It also responds to a national health priority to avail safe-circumcision to men risking foreskin cutting at non-clinical settings in PNG.

## Supporting information

S1 Appendix Focus group guide. (PDF)

S2 Appendix Interview guide 1. (PDF)

S3 Appendix Structured interview questionnaire 1. (PDF)

S4 Appendix Structured interview questionnaire 2. (PDF)

S5 Appendix Interview guide 2. (PDF)

S1 File Phase two survey data. (SAV)

S2 File Phase three survey data. (SAV)
