# Supplementary material for: Re-establishing safer medical-circumcision-integrated initiation ceremonies for HIV prevention in a rural setting in Papua New Guinea. A multi-method acceptability study
Source: PLoS One. 2017 Nov 8;12(11):e0187577. doi: 10.1371/journal.pone.0187577 (PMC5678725; doi:10.1371/journal.pone.0187577)
Supplement: S1 Appendix — (PDF) [file pone.0187577.s001.pdf]

Appendix A

EAST SEPIK PROVINCIAL AIDS COMMITTEE

Faculty of Health Sciences

Focus Group Discussion Guide

Focus Group Number:	Venue:
Date:	Moderator:
Start time:	Note Taker:
End Time:	Supervisor

Project Objective:
To assess the views of community leaders in Yangoru-Sausia regarding traditional best practice especially initiation ceremonies and the possibility of behavior change for HIV prevention among the young people of Yangoru-Sausia District

1. Can you please describe some of the cultural traditional practices especially to do with initiation ceremonies in your area?

2. What was/is the purpose of those cultural practices/initiation ceremonies?

3. Can you please compare traditional cultural ceremonies especially initiation performed in the days of your fathers to the ceremonies performed nowadays?

4. What is your view regarding the behavior/attitude of young people in the days where traditional cultural ceremonies/initiations were vibrant?

5. What is your view regarding the behavior/attitude of young people nowadays?

6. Can you please identify some behavior/attitude of today's young people that is not acceptable?

7. What are the risks of such unacceptable behavior/attitude?

8. Why do you think young people nowadays behave the way they do?

9. What are some ways or methods we could employ to change our young peoples' behavior for the better?

10. Great effort and priority is given to stop HIV/AIDS but the number of infected cases continue to rise. Why do you think this is so?

11. If we are to employ some methods to change our young peoples' behavior, what methods would you recommend for young people of Yangoru-Sausia District?

12. What makes you think that the method you mentioned will have a positive effect on the way young people behave?

13. What is your view regarding this initiative (project)?

END OF DISCUSSION

Subject Information Statement and Consent Form
For Participants

Semi-Structured Interviews

Background and purpose of study

You are invited to participate in this research study by sharing with us your experiences and insights about traditional practices in Yangoru- Sausia and its effects on behavior especially on young people.

This study is conducted by Dr. Clement Manineng of the Faculty of Health Science of Divine Word University in collaboration with East Sepik Provincial AIDS Committee. The study is assisted by Professor Francis Hombhanje and Fr. Dr. Patrick Gesch.

The purpose of the study is to document the views of community leaders /elders in Yangoru-Sausia electorate regarding traditional best practices, a means for behavior change among young people of Yangoru-Sausia, East Sepik Province. It is evident that the current methods for fighting HIV is not working so the findings from this study will be used to inform the government through the National Department of Health about the possibility of incorporating traditional best practices as one of its HIV/AIDS prevention strategies

Description of Study and Risks
If you decide to participate, you will join 9 other community leaders in a group discussion you using a set of pre-defined questions. You are free to make your comments whether it be positive or negative. The discussion will be recorded using a voice recorder so that we do not miss any important things mentioned by you or other leaders. The discussion will take between 30 minutes to 1 hour. Before the discussion you will sign a consent form giving us permission to record your voice as well as to document your views on the topic.

Confidentiality and Disclosure of Information
Your real name will not be used in the study nor will it be used during the interview. Instead, a make up name will be used to protect your identity. Any information collected by the study will remain confidential and will not be disclosed except to the researchers. We plan to present the results at significant health conferences including the PNG Medical Symposium and may also get the work published in relevant journals. The data presented at these meetings will not identify individual subject information.

Benefits of Participation
You will be provided with refreshments for your participation.

Questions
If you have any questions about this study or about your participation, I will answer them now. And if you have questions later, you can contact me on mobile phone: 7672 3116 / 7688 9014

Your Consent
Now having being fully informed about the study, you can now make a decision to participate or not to participate. If you decide not to participate, you are free to withdraw your consent and discontinue your participation at any time without any problems. You will be given a copy of this form to keep.

Dr. Clement Manineng
Divine Word University
Phone: 7672 3116 / 724 31886
Email: cmanineng@dwu.ac.pg

Mr. Emil Trowalle
East Sepik Provincial Aids Committee
Phone: 7674 9563 / 4561 844
Emaile : esphrs@daltron.com.pg

Focus Group Discussion Questionnare on Traditional Best Practices for HIV/AIDS Prevention in Yangoru, Sausia of East Sepik Province.

Consent Form

I ……………………………………………………………….hereby consent to participate in this interview as a participant. I was made aware and fully understand the content and aim of this study and am willing to freely express my views regarding Traditional Best Practices for HIV/AIDS Prevention in Yangoru-Sausia District of East Sepik Province. I also consent to have my voice recorded.

Signature of Participant…………………………………..Date Signed:……………………….

………………………………………. (Signature of witness)

……………………………………. (Name of Witness)
