# Supplementary material for: Re-establishing safer medical-circumcision-integrated initiation ceremonies for HIV prevention in a rural setting in Papua New Guinea. A multi-method acceptability study
Source: PLoS One. 2017 Nov 8;12(11):e0187577. doi: 10.1371/journal.pone.0187577 (PMC5678725; doi:10.1371/journal.pone.0187577)
Supplement: S2 Appendix — (PDF) [file pone.0187577.s002.pdf]

Appendix B

Semi-Structure Interview Guide

Interview Number:	Venue:
Date:	Interviewer:
Time:	Supervisor:

1. **Can you please tell us about the male initiation ceremony in your area?**
   -When is it done?
   -What is the reason for it?
   -How do people prepare for it?
   -What happens at the ceremony?
   -How long does it last?
   -Who is eligible to participate?
   -Who is responsible for organizing it?
   -Is there any risks to the participant?
   -Is there any risk to the initiator?

2. **Can you please tell us about the female initiation ceremony in your area?**
   -When is it done?
   -What is the reason for it?
   -How do people prepare for it?
   -What happens at the ceremony?
   -How long does it last? Initiator
   -Who is eligible to participate?
   -Who is responsible for organizing it?
   -Is there any risks to the participant?
   -Is there any risk to the initiator?

3. **Why is initiation ceremony not practiced today?**

4. **How can initiation ceremonies be revived?**
   -Who can lead?
   -Are people with custom knowledge still alive?
   -What are some taboos to follow to revive such a ceremony?
   -What is required?

5. **Do you think, initiation ceremonies can change the attitude and behavior of young people today?**
   - How can initiation ceremony change behavior?
   - What happens during initiation that favors behavior change

6. **Do you have anything else to say?**

Subject Information Statement and Consent Form
For Participants

Semi-Structured Interviews

Background and purpose of study

You are invited to participate in this research study by sharing with us your experiences and insights about traditional practices in Yangoru- Sausia and its effects on behavior especially on young people.

This study is conducted by Dr. Clement Manineng of the Faculty of Health Science of Divine Word University in collaboration with East Sepik Provincial AIDS Committee. The study is assisted by Professor Francis Hombhanje and Fr. Dr. Patrick Gesch.

The purpose of the study is to document the views of community leaders /elders in Yangoru-Sausia electorate regarding traditional best practices, a means for behavior change among young people of Yangoru-Sausia, East Sepik Province. It is evident that the current methods for fighting HIV is not working so the findings from this study will be used to inform the government through the National Department of Health about the possibility of incorporating traditional best practices as one of its HIV/AIDS prevention strategies

Description of Study and Risks
If you decide to participate, one of the researchers will interview you using a set of pre-defined questions. You are free to make your comments whether it be positive or negative. The interview will be recorded using a voice recorder so that we do not miss any important things mentioned by you. The interview will take between 30 minutes to 1 hour. Before the discussion you will sign a consent form giving us permission to record your voice as well as to document your views on the topic.

Confidentiality and Disclosure of Information
Your real name will not be used in the study nor will it be used during the interview. Instead, a make up name will be used to protect your identity. Any information collected by the study will remain confidential and will not be disclosed except to the researchers. We plan to present the results at significant health conferences including the PNG Medical Symposium and may also get the work published in relevant journals. The data presented at these meetings will not identify individual subject information.

Benefits of Participation
You will be provided with refreshments for your participation.

Questions
If you have any questions about this study or about your participation, I will answer them now. And if you have questions later, you can contact me on mobile phone: 7672 3116 / 7688 9014

Your Consent
Now having being fully informed about the study, you can now make a decision to participate or not to participate. If you decide not to participate, you are free to withdraw your consent and discontinue your participation at any time without any problems. You will be given a copy of this form to keep.

Dr. Clement Manineng	*Mr. Emil Trowalle*
Divine Word University	*East Sepik Provincial Aids Committee*
Phone: 7672 3116 / 724 31886	*Phone: 7674 9563 / 4561 844*
Email: cmanineng@dwu.ac.pg	*Emaile : esphrs@daltron.com.pg*

Semi- structure interview on Traditional Best Practices for HIV/AIDS Prevention in Yangoru, Sausia of East Sepik Province.

Consent Form

I ……………………………………………………………….hereby consent to participate in this interview as a participant. I was made aware and fully understand the content and aim of this study and am willing to freely express my views regarding Traditional Best Practices for HIV/AIDS Prevention in Yangoru-Sausia District of East Sepik Province. I also consent to have my voice recorded.

Signature of Participant…………………………………..Date Signed:……………………….

……………………………………….　　　　……………………………………..
(Signature of witness)　　　　(Name of Witness)
