# Supplementary material for: Re-establishing safer medical-circumcision-integrated initiation ceremonies for HIV prevention in a rural setting in Papua New Guinea. A multi-method acceptability study
Source: PLoS One. 2017 Nov 8;12(11):e0187577. doi: 10.1371/journal.pone.0187577 (PMC5678725; doi:10.1371/journal.pone.0187577)
Supplement: S3 Appendix — (PDF) [file pone.0187577.s003.pdf]

Appendix C

Subject Information Statement and Consent Form For Participants

Structured Interviews

Participant No. ☐ **Self Administered** ☐ **Interviewed** ☐

Background and purpose of study

You are invited to participate in this research study by answering the questions asked by a researcher. The questions will be on traditional practices in Yangoru- Sausia and its effects on behavior especially on young people.

This study is conducted by Dr. Clement Manineng of the Faculty of Health Science of Divine Word University in collaboration with East Sepik Provincial AIDS Committee. The study is assisted by Professor Francis Hombhanje and Fr. Dr. Patrick Gesch.

The purpose of the study is to document the views of community leaders /elders in Yangoru-Sausia electorate regarding traditional best practices, a means for behavior change among young people of Yangoru-Sausia, East Sepik Province. It is evident that the current methods for fighting HIV is not working so the findings from this study will be used to inform the government through the National Department of Health about the possibility of incorporating traditional best practices as one of its HIV/AIDS prevention strategies

Description of Study and Risks

If you decide to participate, one of the researchers will interview you using a set of pre-defined questions. You are free to make your comments whether it be positive or negative. The interview will fill out the questionnaire according to the response you provide for each question. The interview will take between 20-40 minutes. Your response to the questions will be indicative of your consent to participate. If you decide not to participate, you are free to withdraw your consent and discontinue your participation at any time without any problems. You will be given a copy of this form to keep.

Confidentiality and Disclosure of Information

Your real name will not be used in the study nor will it be used during the interview. Instead, a make up name will be used to protect your identity. Any information collected by the study will remain confidential and will not be disclosed except to the researchers. We plan to present the results at significant health conferences including the PNG Medical Symposium and may also get the work published in relevant journals. The data presented at these meetings will not identify individual subject information.

Questions

If you have any questions about this study or about your participation, I will answer them now. And if you have questions later, you can contact one of the two persons below.

Dr. Clement Manineng
Divine Word University
Phone: 7672 3116 / 724 31886
Email: cmanineng@dwu.ac.pg

Mr. Emil Trowale
East Sepik Provincial Aids Committee
Phone: 7674 9563 / 4561 844
Email: epsr@daltron.net.pg

Section One

In this section, you will give us a little information about yourself. Please read the question on your left and circle the number in the middle column that corresponds to your correct answer on the right column. For those sections that do not provide options, please write your answer in the space provided.

1	What is your gender?	1<br>2	Male<br>Female
2	What is your age group?	1<br>2<br>3<br>4<br>5<br>6	20 -25 years<br>25 -35 years<br>35-40 years<br>40 -50 years<br>50 – 60 years<br>> 60 years
3	How many male children do you have?		
4	How many female children to you have?		
5	How old is your oldest child?	1<br>2<br>3<br>4<br>5	<10 years<br>>10 years and <15 years<br>>15 years<br>>25 years<br>Not sure
6	How old is your youngest child?	1<br>2<br>3<br>4<br>5<br>6	<5 years<br><10 years<br>>10 years and <15 years<br>>15 years<br>>25 years<br>Not sure
7	What is the name of your village?		
8	How long have you been in your village?	1<br>2<br>3<br>4	All my life<br>Good part of my life<br>Small part of my life<br>Not sure
9	What is your highest level of education	1<br>2<br>3<br>4<br>5<br>6	University/College<br>National High/Secondary<br>High School<br>Community/Primary School<br>No formal Education<br>Other…………………(specify)
10	What are your sources of income (can circle more then one choice)	1<br>2<br>3<br>4<br>5<br>6<br>7	Formal job<br>Cash crops<br>Food crops<br>Informal business (specify…..)<br>Customary payments<br>Other (specify)……….<br>None
11	If you have a formal job, what is your job (for each job, please describe the exact type of job eg. Health worker = Nursing Officer)	1<br>2<br>3<br>4	Teacher……………………….<br>Policeman……………………..<br>Health worker………………….<br>Local level government

		5<br>6<br>7	worker…………………<br>Church worker (pastor etc)……………………….<br>Tradesman………………….<br>Other (specify)… ……….
12	What is the name of your Tribe?		
13	What is the name of your tribal leader?		
14	Did you ever play a leadership role in your community?	1<br>2<br>3	Yes<br>No<br>Not sure
15	If yes, what was the role you played? (Can choose more than one option)	1<br>2<br>3<br>4<br>5	Counselor<br>Mediator<br>Ceremonial Leader<br>Family Leader<br>Other (specify)…..

Section Two

In this section, we will ask you about your knowledge on HIV. Please read the question on your left and circle the number in the middle column that corresponds to your correct answer on the right column. For those sections that do not provide options, please write your answer in the space provided.

16	What are the different ways by which HIV is transmitted?	1<br>2<br>3<br>4<br>5	Sex<br>Sharing sharps<br>Mother to baby<br>Blood Transfusion<br>Not sure
17	What is the main way by which HIV is transmitted ?(circle one answer)	1<br>2<br>3<br>4<br>5<br>6	Sex<br>Sharing sharps<br>Mother to baby<br>Blood Transfusion<br>Other (specify)……………..<br>Not sure
18	Which group of people are most at risk of getting HIV infection?	1<br>2<br>3<br>4<br>5<br>6	Small children<br>Teenagers<br>Young adults and youths<br>Older adults<br>Other (specify)……………..<br>Not sure
19	Can the use of condom prevent HIV infection?	1<br>2<br>3	Yes<br>No<br>Not sure
20	If no, why is it that condom cannot prevent HIV infection?	1<br>2<br><br>3<br>4<br>5	It is not reliable<br>Has invisible holes that allow virus through<br>Can break easily<br>Other (specify)<br>Not sure
21	Is the promotion of condom useful in the fight against	1	Yes

	HIV?	2<br>3	No<br>Not sure
22	If no, why is it not useful	1<br>2<br><br>3<br><br>4<br>5	Promotes promiscuity<br>Diverts useful resources (expensive)<br>Unreliable (can break and has holes)<br>Other (specify)……………..<br>Not sure
23	List some factors you know of that promote the spread of HIV in your area.	•	
		•	
		•	
		•	
		•	
		•	
		•	
		•	

Section Three

In this section, you will give us information about your experience as well as you views on traditional ceremonies in your area. Please read the question on your left and circle the number in the middle column that corresponds to your correct answer on the right column. For those sections that do not provide options, please write your answer in the space provided.

24	Did you ever participate in a traditional ceremony	1<br>2	Yes<br>No
25	If yes, what was the traditional ceremony you participated in? (can circle more than one options)	1<br>2<br><br>3<br>4<br>5<br>6	Pride price ceremony<br>Initiation ceremony (male/ female)<br>Peace ceremony (friend)<br>Funeral ceremony<br>Other (specify)…..<br>None
26	If No, did you ever witness any of the traditional ceremonies mentioned above?	1<br>2	Yes<br>No
27	If Yes, what were the ceremonies you witnessed? (can circle more than one option	1<br>2<br><br>3<br>4<br>5<br>6<br>7	Pride price ceremony<br>Initiation ceremony (male/ female)<br>Peace ceremony (friend)<br>Funeral ceremony<br>Other (specify)…..<br>None<br>Not sure
28	When was the last male initiation ceremony performed in your area?	1<br>2<br>3<br>4<br>5<br>6	Recently (give year)…….<br>Few years ago (give year)….<br>Many years ago<br>Cannot remember<br>Never had one<br>Not sure

29	When was the last female initiation ceremony performed in your area?	1<br>2<br>3<br>4<br>5<br>6	Recently (give year)…….<br>Few years ago (give year)….<br>Many years ago<br>Cannot remember<br>Never had one<br>Not sure
30	How many initiation ceremonies have you witnessed or heard of in Yangoru-Sausia in the last 10 years	1<br>2<br>3<br>5<br>6	More than10<br>Between 5 and 10<br>Less than 5<br>None<br>Not sure
31	Is initiation ceremony good for your area?	1<br>2<br>3	Yes<br>No<br>Not sure
32	If Yes, in what way is it good? (can circle more than one option)	1<br>2<br>3<br>4<br>5<br>6<br>7	It gives self discipline<br>It gives strength to be an adult<br>It is a source for wisdom<br>It is a form of identity<br>It brings honor<br>Other (specify)…………………<br>Not sure
33	If No, in what way is it not good? (can circle more than once option	1<br>2<br>3<br>4	Health risk<br>Costly<br>Evil<br>Other (specify)………………….<br>Not sure
34	Can initiation ceremonies help the fight against HIV in your area?	1<br>2<br>3	Yes<br>No<br>Not sure
35	If Yes, how can initiation ceremonies help fight HIV in your area? (Can circle more than one option)	1<br>2<br>3<br>4	It gives self discipline<br>It is a source for wisdom<br>Avenue for behavior change<br>Other (specify)………………..
36	Would you like initiation ceremonies to be revived?	1<br>2<br>3	Yes<br>No<br>Not sure
37	If no, why not?	1<br>2<br>3<br>4<br>5	Health risk<br>Costly<br>Evil<br>Other (specify)………………….<br>Not sure

38. Is there anything else you wish to mention?
