# Supplementary material for: Re-establishing safer medical-circumcision-integrated initiation ceremonies for HIV prevention in a rural setting in Papua New Guinea. A multi-method acceptability study
Source: PLoS One. 2017 Nov 8;12(11):e0187577. doi: 10.1371/journal.pone.0187577 (PMC5678725; doi:10.1371/journal.pone.0187577)
Supplement: S4 Appendix — (PDF) [file pone.0187577.s004.pdf]

Integrating Medical Male Circumcision into Initiation Ceremonies in Yangoru-Sausia

Subject Information Statement and Interview form: Cross Sectional Survey

Participant No. ☐ **Interviewed** ☐ **Self-Administered** ☐

Background and purpose of study

You are invited to participate in this research by answering the questions in this interview form. The questions will be about your views on including medical circumcision at traditional male initiation ceremonies in Yangoru- Sausia

This study is being conducted in collaboration with Divine Word University and East Sepik Provincial AIDS Committee. When complete, this study will contribute towards Dr. Clement Manineng's doctoral qualification at James Cook University, Australia.

The overall purpose of this study is to assess whether it is acceptable and feasible for medical circumcision to take place at male initiation ceremonies in Yangoru-Sausia. In order to achieve the overall purpose, this study will assess the local peoples' views, assess the challenges and positives of including medical circumcision at initiation sites and assess what effect the modified ceremony may have had on the initiates and the communities. If the assessment turns out to be positive, the government and other service providers both in PNG and overseas will be informed about the opportunity to include health programs into cultural practices such as male initiation ceremonies both in Yangoru-Sausia and other tribal cultural groups.

Description of Study and Risks

If you decide to participate, a researcher will interview you using an interview form. Alternatively, you can receive an interview form and fill it out yourself. You are free to make your comments whether it be positive or negative. The researcher will fill out the questionnaire (if you prefer to be interviewed) according to the response you provide for each question. The interview will take between 20-40 minutes. If you wish to participate, please provide your consent on the consent form before you proceed to answer the questions. If you decide not to participate, you are free to withdraw your consent and discontinue your participation at any time without any problems. You will be given a copy of this form to keep.

Confidentiality and Disclosure of Information

Your name will not be written in the interview form, nor will it appear in any of our reports or presentations. All information collected during the study will remain confidential and will not be disclosed to anyone apart from the researchers. The results (at the end of the study) will be presented at significant health conferences including the PNG Medical Symposium and also be published in relevant journals. The data presented at these meetings will not identify you as a participant of this study.

Questions

If you have any questions about this study or about your participation, I will answer them now. If you have concerns regarding the ethical conduct of this study, please contact James Cook University's Human Ethics Research Office (contacts provided below).

Human Ethics, Research Office
James Cook University, Townsville, Qld, 4811
Phone:+61 (07) 4781 5011 (ethics@jcu.edu.au)

Section One: Background information<br>In this section, you will give us some information about yourself. Please read the question on your left and circle the number in the middle column that corresponds to your correct answer on the right column. For those sections that do not provide options, please write your answers in the spaces provided.			
1	Are you a man or a woman?	1<br>2	Man<br>Woman
2	How old are you?	I am ______years old	
3	Are you married?	1<br>2<br>3	Yes<br>No (go to question 6)<br>Other__________________
4	If you are married, how many children do you have?	I have____children	
5	If you have children, please specify their sex and age		
6	What church do you belong to?	1<br>2<br>3<br>4	Catholic<br>AOG<br>SDA<br>Other___________________
7	What local level government area (LLG) do you belong to?	1<br>2<br>3<br>4<br>5	Numbo<br>Sause<br>East Yangoru<br>West Yangoru<br>Other_________________
8	How long have you been in Yangoru-Sausia?	For ________years	
9	What is the highest level of formal education you have completed?	1<br>2<br>3<br>4<br>5<br>6	Have not attended school<br>Elementary (prep – elementary 2)<br>Primary (grade 3-8)<br>High School (grade 9-10)<br>Secondary School (grade 11-12)<br>Other (specify __________________
10	What is your <u>*main*</u> source of income?	1<br>2<br>3<br>4<br>5<br>6	Sale of cash crops eg.cocoa<br>Sale of garden crops eg. Banana<br>Formal business eg. Trade store<br>Informal business eg. Sale of betel nut<br>Formal job<br>Other________________________
11	If you have a formal job, please describe your job		
12	Were you initiated in an initiation ceremony in Yangoru-Sausia?	1<br>2<br>3	Yes<br>No<br>Other___________________
13	Are you aware of any initiation ceremony that had happened in Yangoru-Sausia?	1<br>2<br>3	Yes<br>No (go to section two)<br>Other___________________
14	If yes, please provide details of the ceremony	1. male or female<br>2. Venue____________________<br>3. Date______________________<br>4. Other_____________________	

Section Two: HIV Knowledge<br>In this section, we will ask you about your knowledge on HIV.			
15	Can a woman get HIV from having sex with a man who has HIV?	1<br>2<br>3	Yes<br>No<br>Not sure
16	Can a man get HIV from having sex with a woman who has HIV?	1<br>2<br>3	Yes<br>No<br>Not sure
17	Can a man get HIV from having sex with a man who has HIV?	1<br>2<br>3	Yes<br>No<br>Not sure
18	Can a person get HIV from a needle, razor or other cutting tools that have already been used by someone else for tattooing, scarification or circumcision?	1<br>2<br>3	Yes<br>No<br>Not sure
19	Can a person get HIV from mosquito bites?	1<br>2<br>3	Yes<br>No<br>Not sure
20	If someone with HIV coughs or sneezes near another person, can that person get HIV?	1<br>2<br>3	Yes<br>No<br>Not sure
21	Can a person get HIV by hugging someone who has HIV?	1<br>2<br>3	Yes<br>No<br>Not sure
22	Can a person get HIV by sharing food with someone who has HIV?	1<br>2<br>3	Yes<br>No<br>Not sure
23	If a woman with HIV is pregnant, can her baby become infected with HIV?	1<br>2<br>3	Yes<br>No<br>Not sure
24	Can the family planning pill protect a woman from HIV infection?	1<br>2<br>3	Yes<br>No<br>Not sure
25	If condoms are used correctly during sex do they help protect people from getting HIV?	1<br>2<br>3	Yes<br>No<br>Not sure
26	Can someone who looks healthy have HIV?	1<br>2<br>3	Yes<br>No<br>Not sure
**Section Three: Modified initiation ceremony**<br>In this section, you will give us information about you views on the possibility of integrating medical circumcision into male initiation ceremonies in Yangoru-Sausia. Please read the question on your left and circle your answer from the options provided on the right. For those sections that do not provide options, please write your answer in the spaces provided.			
27	Do you think male initiation ceremonies in Yangoru-Sausia can enable young men to become responsible adults?	1<br>2<br>3	Yes<br>No<br>Unsure

28	Please provide one reason (the main reason) for your answer to above question.<br>____<br>____<br>____<br>____<br>____<br>____		
29	Would you agree for health workers to provide medical circumcision (and Voluntary HIV Counselling and Testing) at male initiation ceremonies in Yangoru-Sausia?	1<br>2<br>3	Yes<br>No<br>Unsure
30	Please provide reasons for your answer to above question.<br>____<br>____<br>____<br>____<br>____<br>____		
31	Would you like the male initiation ceremonies in Yangoru-Sausia to be revived?	1<br>2<br>3	Yes<br>No<br>Unsure
32	Please provide reasons for your answer to the above question.____<br>____<br>____<br>____<br>____<br>____<br>____<br>____		
33	Do you have anything else you wish to say?<br>____<br>____<br>____		

*********************Thank you so much for participating in this study. The results of this study will be made available to you and the community through a feedback presentation to be organized soon*********************.
