# Supplementary material for: Re-establishing safer medical-circumcision-integrated initiation ceremonies for HIV prevention in a rural setting in Papua New Guinea. A multi-method acceptability study
Source: PLoS One. 2017 Nov 8;12(11):e0187577. doi: 10.1371/journal.pone.0187577 (PMC5678725; doi:10.1371/journal.pone.0187577)
Supplement: S5 Appendix — (PDF) [file pone.0187577.s005.pdf]

Integrating Medical Male Circumcision into Initiation Ceremonies in Yangoru-Sausia

Interview Guide: Culture Leaders

Open-ended questions

I. What do you think about the Yangoru-Sausia male initiation ceremonies and new things that are coming in such as health workers being involved?

II. What are your opinions about techniques that will come in the future like instruments that can be attached to the foreskin to achieve circumcision? *(a video on the use of pre-pex circumcision device will be shown at this point)*

Prompts

- *Would you be happy with the involvement of health workers at male initiation ceremonies? Please explain.*
- *Can the initiation ceremonies take place today without the involvement of health workers?*
- *Will today's young men be willing to be initiated in the old ways –that is, with urethral bleeding and severe beatings?*
- *What parts of the initiation ceremonies will be affected by the involvement of health workers?*
- *If physical pain must be felt by the initiates, how much physical pain is required? Can physical pain be substituted with another activity?*
- *If blood must be shed by the initiates, how much blood loss is required? Is simple oozing of blood enough or blood needs to spurt out of an initiate's body? Is the amount of blood loss at medical circumcision enough to signify transition from boy to man?*
- *What do you think about the possibility of using circumcision devices at male initiation ceremonies? What are some good sides, what are some bad sides and what is your personal opinion?*
- *Do you have anything else you wish to say?*
